# Natural Killer Cells Exhibit a Peculiar Phenotypic Profile in Systemic Sclerosis and Are Potent Inducers of Endothelial Microparticles Release

**DOI:** 10.3389/fimmu.2018.01665

**Published:** 2018-07-18

**Authors:** Audrey Benyamine, Jérémy Magalon, Florence Sabatier, Luc Lyonnet, Stéphane Robert, Chloé Dumoulin, Sophie Morange, Karin Mazodier, Gilles Kaplanski, Martine Reynaud-Gaubert, Pascal Rossi, Françoise Dignat-George, Brigitte Granel, Pascale Paul

**Affiliations:** ^1^Internal Medicine Department, Pôle MINC, Hôpital Nord, Assistance Publique Hôpitaux de Marseille (AP-HM), Marseille, France; ^2^Aix Marseille Univ, INSERM, INRA, C2VN, Marseille, France; ^3^Cell Therapy Unit, Hôpital de la Conception, AP-HM, INSERM CIC BT 1409, Marseille, France; ^4^Hematology and Vascular Biology Department, Hôpital de la Conception, AP-HM, Marseille, France; ^5^Centre d’Investigation clinique (CIC), Hôpital de la Conception, AP-HM, Marseille, France; ^6^Internal Medicine and Clinical Immunology Department, Pôle MINC, Hôpital de la Conception, AP-HM, Marseille, France; ^7^CHU Nord, Pneumology Department, APHM, Marseille, France

**Keywords:** natural killer cells, endothelial microparticles, fractalkine, CX3CR1, systemic sclerosis

## Abstract

The pathophysiology of systemic sclerosis (SSc) involves early endothelial and immune activation, both preceding the onset of fibrosis. We previously identified soluble fractalkine and circulating endothelial microparticles (EMPs) as biomarkers of endothelial inflammatory activation in SSc. Fractalkine plays a dual role as a membrane-bound adhesion molecule expressed in inflamed endothelial cells (ECs) and as a chemokine involved in the recruitment, transmigration, and cytotoxic activation of immune cells that express CX3CR1, the receptor of fractalkine, namely CD8 and γδ T cells and natural killer (NK) cells. We aimed to quantify circulating cytotoxic immune cells and their expression of CX3CR1. We further investigated the expression profile of NK cells chemokine receptors and activation markers and the potential of NK cells to induce EC activation in SSc. We performed a monocentric study (NCT 02636127) enrolling 15 SSc patients [15 females, median age of 55 years (39–63), 11 limited cutaneous form and 4 diffuse] and 15 healthy controls. Serum fractalkine levels were significantly increased in SSc patients. Circulating CD8 T cells numbers were decreased in SSc patients with no difference in their CX3CR1 expression. Circulating γδ T cells and NK cells numbers were preserved. CX3CR1 expression in CD8 and γδ T cells did not differ between SSc patients and controls. The percentage and level of CX3CR1 expression in NK cells were significantly lowered in SSc patients. Percentages of CXCR4, NKG2D, CD69-expressing NK cells, and their expression levels were decreased in NK cells. Conversely, CD16 level expression and percentages of CD16^+^ NK cells were preserved. The exposure of human microvascular dermic EC line (HMVEC-d) to peripheral blood mononuclear cells resulted in similar NK cells degranulation activity in SSc patients and controls. We further showed that NK cells purified from the blood of SSc patients induced enhanced release of EMPs than NK cells from controls. This study evidenced a peculiar NK cells phenotype in SSc characterized by decreased chemokine and activation receptors expression, that might reflect NK cells involvement in the pathogenic process. It also highlighted the role of NK cells as a potent mechanism inducing endothelial activation through enhanced EMPs release.

## Introduction

Systemic sclerosis (SSc) is a complex systemic autoimmune disease characterized by small vessel vasculopathy and multi-organ fibrosis with a lung involvement, highly related to morbidity and mortality (1). The damage of endothelial cells (ECs) is believed to be an early event in the natural history of SSc and may involve environmental, genetic factors, and excessive innate immune responses (2). The disruption of vascular integrity and the subsequent acquisition of an activated endothelial phenotype favor the local recruitment of activated leukocytes and sustain the development of the vasculopathy and tissue fibrosis. Activated immune cells also release soluble inflammatory cytokines and pro-fibrotic growth factors that further activate SSc fibroblasts. The cross talk between activated ECs, fibroblasts, and immune effectors is a major mechanism underlying the pathogenesis and clinical progression of the disease (3–6). Therefore, a better understanding of the quantitative and functional alterations of immune cells is needed in order to shed light on the still unclear pathogenic processes.

Natural killer (NK) cells and γδ T lymphocytes are at the boundary between innate and adaptive immunity (7–9). They are key cytotoxic effectors of the anti-infectious and anti-tumor defenses. Both NK cells and γδ T cells comprise various subsets. Among NK cells, CD56^dim^ cells are the main cytotoxic circulating subset whereas CD56^bright^ NK cells are rather proinflammatory and tissue-trafficking cells (10, 11). Regarding γδ T cells, Vδ2 T cells is the main circulating subset whereas Vδ1 subset is mainly intra-epithelial. NK cells and γδ T cells share expression of non-rearranged activating receptors such as DNAM-1 and NKG2D that sense ligands overexpressed by stressed cells. The antibody-dependent cell-mediated cytotoxicity (ADCC) mechanism can be triggered in NK cells and γδ T cells, after engagement of the CD16 receptor (FcγRIIIa) by the Fc fragment of immunoglobulins. These cells also share non-MHC restricted cytotoxic activity that involve the perforin/granzyme pathway, Fas/Fas ligands interactions, ADCC, and the release of inflammatory cytokines.

Natural killer cells subsets are further defined by their expression of chemokine receptors which influences NK cell migration toward organs. Chemokine receptors are modulated during NK cell activation and contribute to NK cell homeostasis (12). Among them, NK cells express CXCR3 which can bind various proinflammatory chemokines including CXCL4, a biomarker of SSc (13). They also express CXCR4 that specifically binds to stromal-derived-factor 1 SDF-1 (CXCL12) (14, 15). NK cells and to a lesser extent γδ T cells and CD8 T cells express the receptor for its sole ligand fractalkine (CX3CL1) (16). Fractalkine is both a soluble chemokine released after metalloproteases cleavage and an endothelial membrane-bound adhesion molecule. Hence, CX3CR1 is both a receptor that enables the migration of NK cells in response to fractalkine chemokine and an adhesion receptor that can mediate the activation and transmigration of NK cells when binding endothelial membrane-bound fractalkine (16). Interestingly, the endothelial expression and secretion of CX3CL1/fractalkine have been identified as major triggers favoring the recruitment of mononuclear cells expressing CX3CR1 in the affected skin and lung tissue of patients with SSc (17). Variations in the gene encoding the CX3CR1 have been identified as individual susceptibility factors associated with SSc and SSc-pulmonary arterial hypertension (PAH) (18). Furthermore, the disruption of the interaction between fractalkine and CX3CR1 has been shown to dampen the fibrosis process in a murine model of cytokine-induced SSc (19). The identification of the fractalkine/CX3CR1 pathway in SSc pathogenesis thus offers new perspectives for targeted therapy that could limit the inflammatory fibrosis and immune-mediated vascular injury (20).

The release of soluble factors (21–26) and endothelial microparticles (EMPs) (21, 27, 28) have been shown to reflect endothelial injury results (29). We recently identified increased circulating levels of fractalkine and EMPs as a signature of endothelial inflammatory activation and disrupted homeostasis in SSc patients with potential value as a biomarker of organ involvement and disease severity (21). EMPs are also considered as “miniature cells” loaded with bioactive molecules (RNA, proteins, cytokines, and lipids) with a key role in the regulation of intercellular cross talks that sustain inflammation, hemostasis, angiogenesis, and fibrosis (30–32). These EMPs have been shown to be associated with skin and lung fibrosis (27) and to be deleterious *via* the induction of an oxidative burst in a murine model of SSc (33). However, the mechanisms that drive this EMPs release remain poorly understood. The recruitment and activation of NK cells toward target vessel wall and NK cells-mediated microvascular injury were suggested in the pathogenesis in autoimmune vasculitis (34). Interestingly, we recently identified that NK cells are major providers of inflammatory cytokines and endotheliotoxic effects associated with antibody-mediated vasculopathy (35) and impairment of endothelial progenitor cell regenerative functions (36).

Our study thus aimed to investigate the features of NK cells and their potential role as cytotoxic effectors of EC activation and damage in SSc.

## Materials and Methods

### Patients

We performed a monocentric study (NCT 02636127). Fifteen patients with SSc were recruited in the Department of Internal Medicine of Marseilles. All the enrolled patients had a score ≥9 according to the 2013 EULAR/ACR 2013 criteria for SSc (37). The patients were not treated with immunosuppressive drugs except for low-dose steroids under 10 mg/day. Among patients, there were 15 women with a median age of 55 years (39–63 years) (Table 1). Age-matched healthy volunteers (*n* = 15) were recruited as controls. The control group consisted of 15 women with a median age of 54 years (40–61). This study was carried out in accordance with the recommendations of French directives regarding Biomedical Research and the local ethics committee review board of Marseilles, “Comité de Protection des Personnes Sud Méditerranée.” The protocol was approved by the “Comité de Protection des Personnes Sud Méditerranée.” All subjects gave written informed consent in accordance with the Declaration of Helsinki.

**Table 1:** Characteristics of the patients.

	SSc patients (*n* = 15)
Male/female	0/15
Age (years), median ± IQR	55 (39–63)
Cutaneous form: limited/diffuse	11/4
Anti-centromere/anti-topoisomerase I auto-antibodies	7/6
mRSS	8.5 (3–27)
Disease duration ≤3 years/>3 years	5/10
Medsger Severity Scale	
Grade 0–1–2 versus Grade 3–4	0–4–5 versus 2–4
Pitting scars	10
Digital ulcers	5
Telangiectasias	10
Pulmonary function tests	
– TLCO (%)	57 (45–71)
– TLCO/VA (%)	67.8 (56.1–76.5)
– FVC (%)	100.1 (68.38–111.8)
Lung fibrosis on HRCT scan	4
Pulmonary arterial fibrosis	3

*Quantitative variables are described using median and IQR (first quartile–third quartile)*.

*mRSS, modified Rodnan sclerosis score; DLCO, diffusing capacity of the lung for carbon monoxide; FVC, forced vital capacity; VA, alveolar volume; HRCT, high resolution computed tomography; IQR, interquartile range; SSc, systemic sclerosis*.

### Clinical and Standard Biological Assessment

All patients had a physical examination and underwent a blood sample. Clinical characteristics of the study population are summarized in Table 1.

Systemic sclerosis patients were classified as having limited cutaneous SSc or diffuse cutaneous SSc according to the criteria established by LeRoy et al. (38). The disease duration, the presence of Raynaud’s phenomenon, pitting scars, digital ulcers, digital gangrene, or telangiectasias were recorded. The modified Rodnan Skin thickness score (mRSS) was graded on a scale of 0–3 with a maximum total score of 51. Disease severity was measured on a scale of 0–4 according to Medsger’s severity scale (39).

Pulmonary involvement was determined by pulmonary function tests including forced vital capacity and diffusing lung capacity for carbon monoxide (DLCO) and DLCO divided by alveolar volume (DLCO/VA). Fibrosis was diagnosed on Chest Computed Tomography imaging with qualitative criteria consisting in the presence of honey combing, ground glass opacities, reticular abnormalities, traction bronchiectasis, and septa thickening. Systolic pulmonary arterial pressure was measured by transthoracic echocardiography and pulmonary hypertension was confirmed by right heart catheterization.

Antinuclear antibodies were assessed by Indirect Immunofluorescence analysis on HEp-2 cells. Anti-centromere and anti-topoisomerase I antibodies were measured by EliA.

### Cell Culture

Peripheral blood mononuclear cells (PBMCs) from SSc patients and healthy controls were isolated by density gradient centrifugation (Lymphocyte Separation Medium, Abcys, Eurobio).

Freshly isolated PBMCs were stained with monoclonal antibodies for flow cytometry analysis and the remaining PBMCs were frozen until they were used for functional tests.

Natural killer cells were isolated using StemSep Column-Based Cell Isolation kit (StemCell Technologies). The purity of NK cells assessed was determined by flow cytometry and average purity was 93.4%.

Human adult dermal microvascular endothelial cells (HMVEC-d) were obtained from Lonza (Passage 3). They were cultured with EC Growth Medium-MicroVascular (EGM2-MV) (Lonza) and used at Passage 5 for functional tests.

### Flow Cytometry Analysis

2 × 10^5^ PBMCs were washed in Dulbecco’s Phosphate-Buffered Saline (DPBS, Gibco, Thermofisher) and incubated at 4°C for 20 min with specified mAb. Following incubation and washing, samples were analyzed on NAVIOS-3 lasers instrument (Beckman Coulter, Miami, FL, USA), using Kaluza software.

Cytotoxic cell subsets were gated among human PBMCs by flow cytometry using CD45 Krome Orange (Clone J33, Beckman Coulter), CD3 ECD (clone UCHT1, Beckman Coulter), CD56 PC7 (clone NKH-1, Beckman Coulter), CD8 Pacific Blue (clone B9.11, Beckman Coulter), Anti-TCR Pan γ/δ PC5 (clone IMMU510, Beckman Coulter), Anti-TCR Pan α/β PC5 (clone BMA031), Anti-TCR Vδ2-Pacific Blue (clone IMMU389, Beckman Coulter), and Anti-TCR Vδ1 Fitc (clone TS8.2, Thermo Scientific).

The percentage of positive cells and their levels of expression for the following markers were studied with the following labeled antibodies: CX3CR1-PE (clone 2A9, Medical and Biological Laboratories), CD184 (CXCR4)-PE (clone 12G5, Beckman Coulter), CD183 (CXCR3)-Alexa Fluor 488 (clone #49801, R and D systems), CD314 (NKG2D)-PE (clone ON72, Beckman Coulter), CD226 (DNAM1)-Fitc (clone DX11, Becton Dickinson), CD69 PE (clone TP1.55.3, Beckman Coulter), CD16-APC-AlexaFluor750 (clone 3G8, Beckman Coulter), CD107a (LAMP-1)-Fitc (clone H4A3, Becton Dickinson), CD107b (LAMP-2)-Fitc (clone H4B4, Becton Dickinson), and their matched-control istotypes IgG2a PE, IgG1 PE, IgG1 Fitc, IgG1 APC AlexaFluor750.

### CD107 Degranulation Assay

HMVEC-d were cultured overnight in 24 well-plates (2 × 10^5^ cells/well) with Endothelial Basal Medium (EBM2, Lonza) supplemented with either 25% fetal bovine serum (FBS) (Gibco, Thermofisher) or 25% FBS plus IFNγ (Tebu-bio, 50 ng/ml) and TNFα (Euromedex, 20 ng/ml) or 25% healthy control serum or 25% SSc patient serum. All sera were heat inactivated prior to experiments.

Residual rabbit anti-thymocyte globulins (ATG) were obtained from the residual samples that could not be used in clinic. ATG (50 µg/ml) was added to HMVEC-d during 15 min at 37°C, then removed. Cells were washed with DPBS before the addition of PBMCs.

For analysis of CD107 expression, thawed PBMCs (5 × 10^5^ cells/well) were added to HMVEC-d target cells after the overnight culture and incubated at 37°C in the presence of anti-CD107a/b antibodies. After 4 h, PBMCs were collected, washed in PBS and the level of CD107a/b expression was measured by flow cytometry among viable NK cells, defined as CD45^+^DAPI^−^CD3^−^CD56^+^ cells.

### Enumeration of EMPs Release by Flow Cytometry

For EMPs release tests, HMVEC-d were cultured overnight in 96 well-plates (50,000 cells/well) with Endothelial Basal Medium (EBM2, Lonza) supplemented with either 25% FBS (Gibco, Thermofisher) or 25% FBS plus IFNγ (Tebu-bio, 50 ng/ml) and TNFα (Euromedex, 20 ng/ml) or 25% healthy control serum or 25% SSc patient serum. All sera were heat inactivated prior to experiments.

1 × 10^5^ PBMCs or 2 × 10^5^ purified NK cells of SSc patients or age-matched healthy controls were added to the overnight culture in the conditions containing their autologous serum or healthy control serum and conversely.

Supernatants were collected after the overnight culture and were subjected to two successive centrifugations (2,500 *g* for 15 min at room temperature) to remove dead cells and debris-like apoptotic bodies. Annexin V-Fitc and fluorescent antibody reagents were procured from Beckman Coulter (Villepinte, France): CD54 (ICAM-1) PE (clone 84H10), CD45 PC7 (clone J.33).

Endothelial microparticles were enumerated by high sensitivity flow cytometry following standardization as previously described (40, 41). 30 µl of supernatant was incubated with the appropriate amount of specific antibody plus 10 µl of Annexin V-Fitc. Each stained sample was analyzed on CytoFLEX cytometer (Beckman Coulter). Briefly, a standardized side scatter (SSC) microparticle gate was defined using megamix + forward scater (FSC) and SSC beads. Lower limit was defined in SSC just above 0.16 µm bead and upper limit integrated all 0.5 µm bead, still in SSC. This gate is equivalent to a 0.3–1 μm FSC gate, allowing a standardized analysis of small vesicles below 1 µm. Fluorescence gains of CytoFLEX were optimized using sphero 8 peaks beads. EMPs were defined as Annexin V^+^/ICAM1^+^CD45^−^ events. The absolute EMP counts (events per μl) were determined using volume measure provided by the instrument (use of a peristaltic pump). The percentage of increase of EMPs was expressed in reference to the medium condition (EBM2 + 25% FBS).

### Analysis of Soluble Fractalkine and IL-6 Levels

Circulating levels of sfractalkine (CX3CL1) were measured in serum using commercially available ELISA kits from R&D System Inc. (Minneapolis, MN, USA). IL-6 levels were measured in culture supernatants using Human Cytokine/Chemokine Magnetic Bead Panel (Milliplex, Millipore, MO, USA). Assays were performed according to the manufacturer’s instructions.

### Statistical Analysis

Results were expressed as median ± interquartile range (25th–75th percentile, IQR). Statistical analyses were performed using Spearman correlation, Wilcoxon test, and Mann–Whitney *U* test. *p* Values <0.05 were considered significant. Analyses were performed using GraphPad Prism program version 5.

## Results

### Decreased CD8 T Cells but Conserved Numbers of NK Cells and γδ T Cells Circulating Cytotoxic Immune Cells in SSc Patients

**Figure 1:**
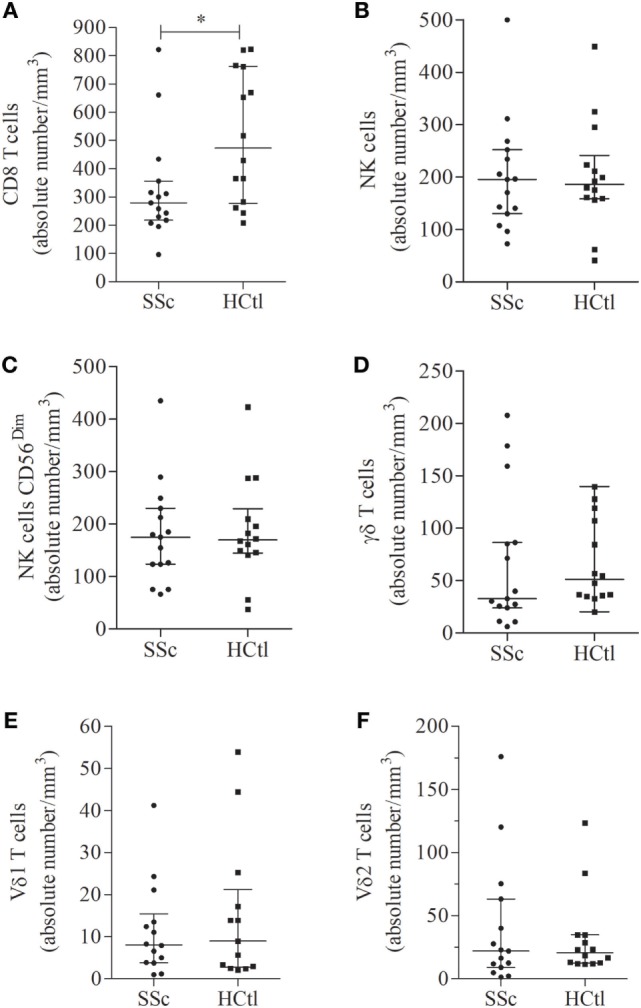
Number of circulating cytotoxic immune cells in systemic sclerosis (SSc) patients. Percentages of circulating immune cells were assessed in the peripheral blood of SSc patients (*n* = 15) with flow cytometry and compared with healthy controls (HCtl) (*n* = 14). Absolute numbers of circulating cells including CD8 T cells **(A)**; natural killer (NK) cells **(B)**; NK cells CD56^dim^ **(C)**; γδ T cells **(D)**; Vδ1 **(E)**; and Vδ2 T cells **(F)** were obtained based on the white blood lymphocyte count and expressed as number of cells per mm^3^. Results were expressed as median ± interquartile range. Statistical difference was established using Mann–Whitney *U* test. *p < 0.05.

We first aimed to determine whether absolute numbers of circulating cytotoxic immune cells, namely CD8 T cells, NK cells, or γδ T cells, were affected in SSc patients. We found that the median number and percentage of circulating CD8 T cells were significantly decreased in SSc patients [279/mm^3^ (218–356) and 19% of leukocytes (13–21)] compared with healthy controls [473.5 (278–763) and 26% (18–33); *p* = 0.0246 and *p* = 0.0142] (Figure 1A). The numbers of circulating NK cells (Figure 1B), NK cells CD56^dim^ (Figure 1C), γδ T cells (Figure 1D), and γδ T cells subpopulations, Vδ1 (Figure 1E) and Vδ2 T cells (Figure 1F), as well as their percentages (*data not shown*) were similar between SSc patients and healthy controls.

### Increased Fractalkine Soluble Levels and Lower Expression of Fractalkine Receptor CX3CR1 Expression in NK Cells from SSc Patients

**Figure 2:**
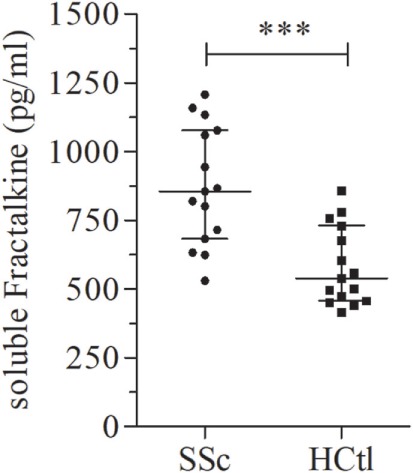
Soluble fractalkine levels in systemic sclerosis (SSc) patients. Soluble fractalkine levels were quantified with ELISA, in the serum of SSc patients (*n* = 15) and healthy controls (HCtl) (*n* = 15). Results were expressed as median ± interquartile range and statistical significance was established using the non-parametric Mann–Whitney *U* test. ***p < 0.001.

As we and others previously demonstrated soluble fractalkine to be a marker of endothelial inflammation and severity of SSc, we aimed to assess fractalkine levels in the studied population. Here, we found that patients with SSc had significantly higher median levels of fractalkine [855.2 pg/ml (683.1–1,077)] than healthy controls [539.3 pg/ml (457.6–731); *p* = 0.0006] (Figure 2).

**Figure 3:**
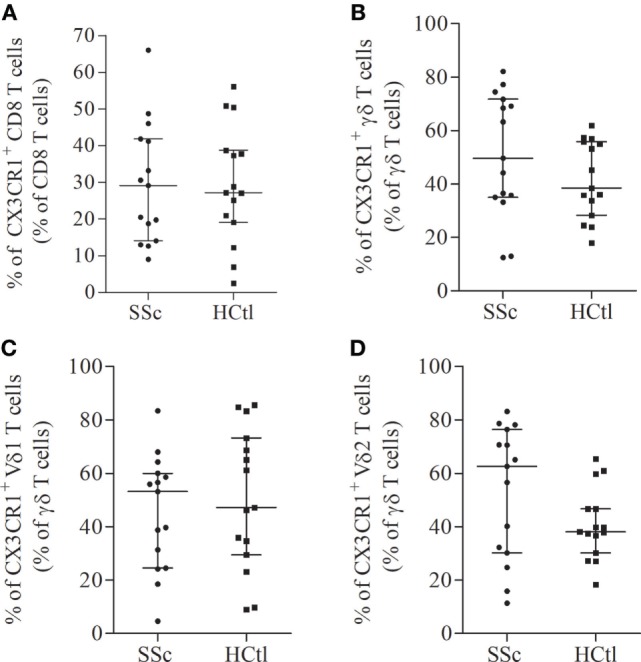
CX3CR1 expression in circulating cytotoxic immune cells from systemic sclerosis (SSc) patients. Percentages of CX3CR1 expressing immune cells were evaluated with flow cytometry analysis on the surface of CD8 T cells **(A)**; γδ T cells **(B)**; Vδ1 **(C)**; and Vδ2 T cells **(D)** from SSc patients (*n* = 15) in comparison with healthy controls (HCtl) (*n* = 15). Results were expressed as median percentages and median ± interquartile range.

**Figure 4:**
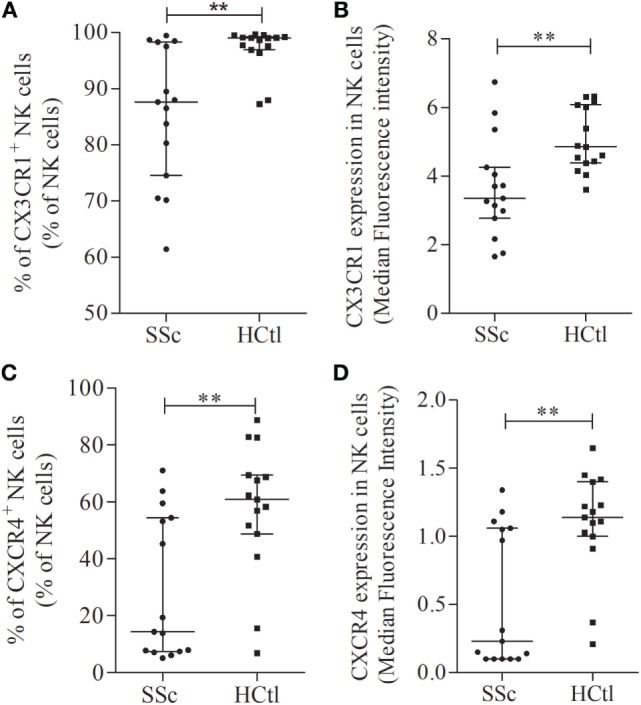
Chemokine receptors expression in natural killer (NK) cells in systemic sclerosis (SSc) patients. CX3CR1 **(A,B)** and CXCR4 expression levels and percentages **(C,D)** were assessed with flow cytometry analysis in whole NK cell from SSc patients (*n* = 15) in comparison with healthy controls (HCtl) (*n* = 15). Results were expressed as percentages of positive cells among NK cells **(A,C)** and median fluorescence intensity **(B,D)**. Results were depicted as median ± interquartile range. Statistical difference was established using Mann–Whitney *U* test. **p < 0.005.

Next, we assessed the surface expression of CX3CR1 namely fractalkine receptor, within circulating cytotoxic immune cell subsets of SSc patients and controls. We first analyzed the percentages of CX3CR1^+^ cytotoxic immune T cell subsets. The proportion of CX3CR1^+^ CD8 T cells (Figure 3A) and γδ T cell subsets (Figure 3B) including Vδ1 (Figure 3C) and Vδ2 T cells (Figure 3D) were comparable in both groups. The percentage of CX3CR1^+^ NK cells was significantly lower in SSc patients [87.63% (74.54–98.28)] than in healthy controls [99.03% (96.95–99.21), *p* = 0.0023] (Figure 4A).

The level of CX3CR1 surface expression of circulating CD8 T cells assessed by flow cytometry with the median fluorescence intensity (MFI) did not differ between SSc patients and healthy controls. CX3CR1 expression was significantly lower in SSc NK cells: [MFI of 4.01 (3.03–7.4)] compared to values observed in healthy controls, [MFI of 5 (4.46–6.13); *p* = 0.0225; Figure 4B]. No statistical difference was noted regarding CX3CR1 expression in γδ T cells, the Vδ1 and Vδ2 T cell subsets (*data not shown*).

While no correlation was noted between circulating fractalkine levels and percentages of CX3CR1 expressing NK cells, CD8 T cells, γδ T cells, and Vδ2 T cells, fractalkine levels were inversely correlated with the percentage of CX3CR1^+^ Vδ1 T cells in SSc patients (*r_S_* = −0.55; *p* = 0.0337).

Collectively, our data show in SSc patients a lower proportion of CX3CR1^+^ NK cells subsets and a downregulation of CX3CR1 on NK cells.

### Decreased Expression of CXCR4 Chemokine Receptor in NK Cells from SSc Patients

We sought to assess the expression of CXCR3 and CXCR4 expression in NK cells from SSc patients and healthy controls.

No statistical difference was noted regarding CXCR3 level of expression and percentages of CXCR3^+^ NK cells between patients and controls (*data not shown*).

The percentage of CXCR4^+^ NK cells [14.36% (7.49–54.45) in SSc versus 60.9% (48.79–69.44) in SSc; *p* = 0.009; Figure 4C], and CXCR4 expression level at the surface of NK cells [0.23 (0.1–1.1) in SSc versus 1.14 (1–1.4); *p* = 0.0032; Figure 4D] were significantly lower in patients than in controls.

### Decreased NKG2D and CD69 Expression and Conserved DNAM1 Expression in NK Cells of SSc Patients

We next further characterized the phenotype of NK cells from SSc patients according to their expression of the NKG2D and DNAM1 activating receptors and of CD69 and CD16 stimulatory/activation receptors.

We observed that percentages of NKG2D^+^, DNAM-1^+^, CD16^+^, CD69^+^ NK cells were similar between patients and healthy controls (*data not shown*).

**Figure 5:**
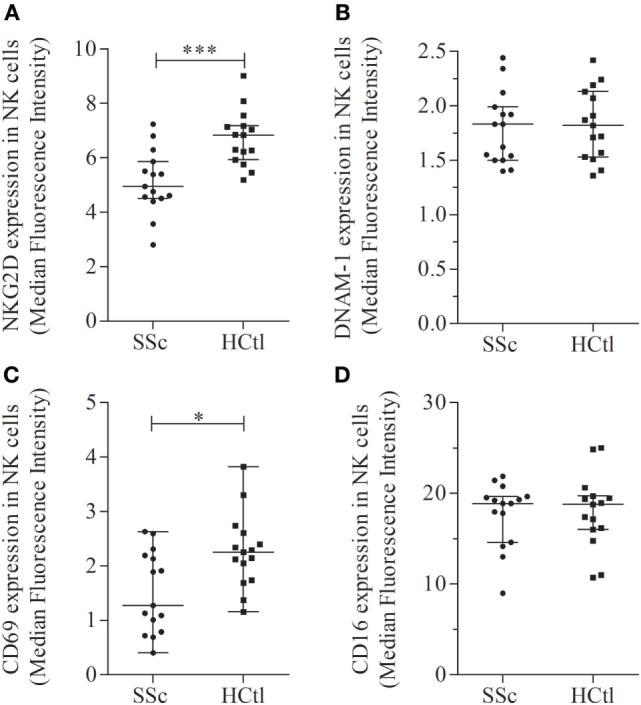
Activation receptors and markers in natural killer (NK) cells of systemic sclerosis (SSc) patients. NKG2D **(A)**, DNAM-1 **(B)**, CD69 **(C)**, and CD16 **(D)** expression levels were evaluated with flow cytometry analysis of NK cells among peripheral blood mononuclear cells from SSc patients and healthy controls (HCtl). Results were expressed as median fluorescence intensity (MFI) and median ± interquartile range. Statistical difference was established using Mann–Whitney *U* test. *p < 0.05; ***p < 0.001.

The level of NKG2D expression was significantly lower in NK cells from SSc patients in comparison with healthy controls [MFI of 5.15 (4.68–6.03) in SSc patients versus 6.92 (5.95–7.27) in healthy controls; *p* = 0.0009] (Figure 5A). The level of DNAM1 expression (Figure 5B) was not statistically different between patients and controls. However, DNAM1 expression was inversely correlated with TLCO in SSc patients (*r_S_* = 0.66; *p* = 0.0368).

The level of CD69 expression was significantly decreased in SSc patients [1.27 (0.79–2.19)] compared with healthy controls [2.25 (1.74–2.61); *p* = 0.0161] (Figure 5C). The level of CD16 expression was similar between SSc patients and controls (Figure 5D).

Collectively, our data demonstrate a peculiar NK cell phenotype in SSc patients characterized by (i) a decreased expression of CX3CR1 and CXCR4 in NK cells (percentages and intensity of expression) and (ii) a lower expression level of the NKG2D activating receptor and of the CD69 activation marker.

### Clinical Association Between Expression Percentages of CX3CR1^+^ and CXCR4^+^ NK Cells and Clinical Characteristics of the Patients

A high heterogeneity was observed among SSc patients regarding CX3CR1 (Figures 4A,B) and CXCR4 expression on NK cells (Figures 4C,D). Hence, we set up to assess whether clinical characteristics of SSc patients were different between patients with high or low CX3CR1 and CXCR4 expression. When patients were segregated in two groups defined by the median percentage of CX3CR1 expressing NK cells (87.63%), a higher frequency of pulmonary fibrosis identified by Chest High Resolution Computed Tomography (*p* = 0.0289) and anti-topoisomerase 1 antibodies (*p* = 0.0483) was observed in patients with the higher percentages of CX3CR1^+^ NK cells.

Conversely, when patients were segregated using median percentage of expression of CXCR4 on NK cells (9.65%), the clinical characteristics of SSc could not be associated with percentages of CXCR4^+^ NK cells.

### Conserved Ability of NK Cells to Degranulate Toward Microvascular Endothelial Target

Natural killer cells from SSc patients were previously found to have either normal of decreased natural cytotoxic activity toward the erythroleukemic cell line K-562. However, few studies have investigated their ability to degranulate toward EC targets.

Here, we used a human dermal microvascular EC line (HMVEC-d) as a target to investigate the NK cell natural cytotoxicity. For this, we assessed NK cell ability to degranulate, after exposure of PBMCs from SSc patients and healthy controls to HMVEC-d targets, in the presence of sera from SSc patients or healthy controls. The levels of exocytosis of CD107a^+^ and CD107b^+^ cytotoxic granules in NK cells were analyzed through multiparametric flow cytometry analysis, within the CD3^−^CD56^+^ NK cell subset gated among CD45^+^ PBMCs.

In addition, we evaluated the ADCC potential of NK cells from SSc patients and controls in response to thymoglobulin (ATG)-coated HMVEC-d targets.

**Figure 6:**
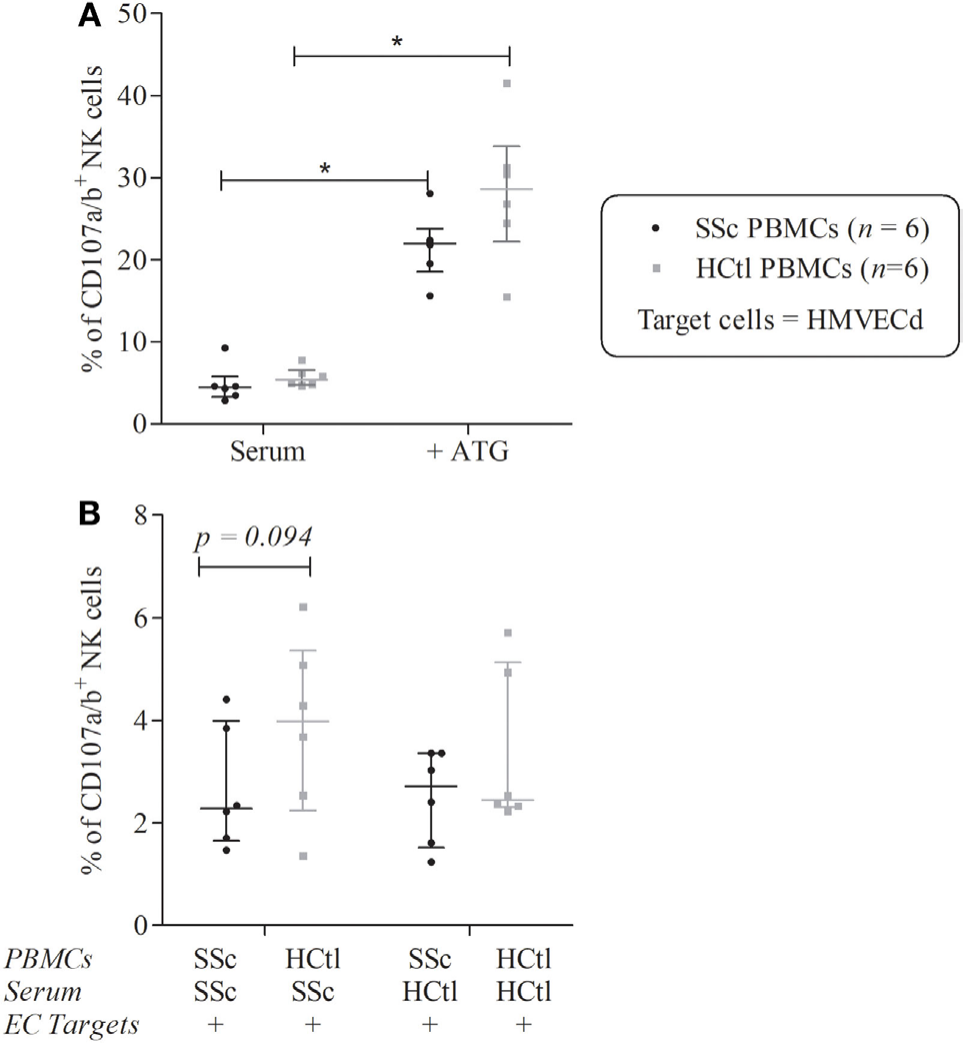
Natural killer (NK) cells degranulation of systemic sclerosis (SSc) patients toward microvascular endothelial cell (EC) target. Cytolytic degranulation of NK cells was assessed by flow cytometry analysis of CD107a/b expression of NK cells among peripheral blood mononuclear cells (PBMCs) after a 4-h coculture with human microvascular dermal ECs (HMVEC-d) with an effector/target ratio of 2/1. **(A)** PBMCs from patients with SSc (*n* = 6) and healthy controls (HCtl) (*n* = 6) were cultured with HMVEC-d with or without thymoglobulin (ATG). **(B)** PBMCs from SSc patients (*n* = 6) and HCtl (*n* = 6) were cultured with HMVEC-d and either SSc serum or HCtl serum and conversely. Cumulative data from five independent experiments. Results were expressed as median percentages ± interquartile range. Statistical significance was established using the non-parametric paired Wilcoxon *U*-test. *p < 0.05.

The degranulation potential of NK cells from both patients and controls was significantly enhanced in response to ATG [% of CD107^+^ SSc NK cells of 22 (18.56–23.82) versus 4.46 (3.3–5.78); *p* = 0.0313; % of CD107^+^ healthy controls NK cells of 28.59 (22.23–33.81) versus 5.4 (4.8–6.59); *p* = 0.0313], whereas NK cells ADCC potentials were similar when using NK cells from patients or healthy controls (*p* = 0.1563) (Figure 6A).

Peripheral blood mononuclear cells from SSc patients were then cocultured with either autologous serum or with serum of healthy controls and conversely. We observed a trend for a lower ability of degranulation of NK cells from SSc compared with healthy donors in the presence of serum of SSc patients (*p* = 0.0938) (Figure 6B).

### Increased EMPs Release Induced by NK Cells from SSc Patients

As NK cells from SSc patients exhibited a specific phenotype, we aimed to assess the functional consequence of such phenotype upon microvascular ECs activation. In order to evaluate endothelial activation and injury, we monitored the EMPs release from HMVEC-d. EMPs were gated as described above and the AnnexinV^+^, CD45^−^, ICAM1^+^ EMPs were quantified.

**Figure 7:**
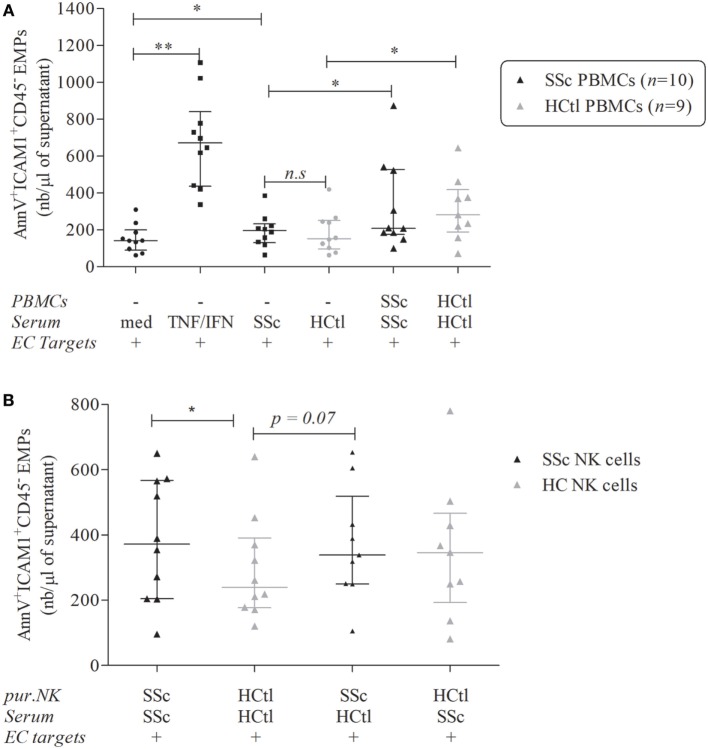
Endothelial microparticles (EMPs) release from microvascular endothelial cells (ECs) induced by natural killer (NK) cells from systemic sclerosis (SSc) patients. EMPs were obtained from the supernatant of overnight-cultured human microvascular dermal EC (HMVEC-d) line. **(A)** HMVEC-d were used as ECs targets and cultured with medium (med), i.e., EBM2 + 25% FCS ± IFNγ (50 ng/ml)/TNFα (20 ng/ml) or serum of SSc patients or healthy controls (HCtl) ± their autologous peripheral blood mononuclear cells (PBMCs) (*n* = 10 and *n* = 9, respectively) with a PBMCs/HMVEC-d target ratio of 1/1. **(B)** HMVEC-d were cultured with EBM2 medium with serum from SSc patients or healthy controls and purified NK cells (pur. NK) from SSc patients (*n* = 10) or healthy controls (*n* = 9–10) with a NK cells/HMVEC-d ratio of 2/1. Results were expressed as median number of EMPs per microliter ± interquartile range. Cumulative data from 10 independent experiments. Statistical significance was established using the non-parametric paired Wilcoxon *U*-test. *p < 0.05, **p < 0.005.

As expected, the overnight culture of HMVEC-d with IFNγ and TNFα significantly boosted their release of EMPs [671.7 (436.2–839.9) versus 141.6 (90.5–199); *p* = 0.002] (Figure 7A).

Exposure of HMVEC-d to serum of SSc patients resulted in higher endothelial EMPs release than when the same cells were exposed with FBS [196.1 (130.4–233) versus 141.6 (90.5–199); *p* = 0.0273] but no significant difference was noted when comparing the effect of serum from healthy controls or SSc patients (Figure 7A).

The addition of PBMCs either obtained from healthy controls or SSc patients increased HMVEC-d-derived EMPs release when compared to the sole addition of autologous sera [281.1 (188–418.7) versus 152.1 (95.94–250.2); *p* = 0.0195 for healthy controls; 208.4 (175.5–526.7) versus 196.1 (130.4–233); *p* = 0.0371 for SSc patients] (Figure 7A). However, no statistical difference was noted between the induction of EMPs by PBMCs from SSc patients and healthy donors (Figure 7A).

We further assessed the specific effect of purified peripheral NK cells isolated from SSc patients compared with healthy controls. The coculture of HMVEC-d with purified NK cells from SSc patients in the presence of autologous SSc serum was associated with more EMPs release than NK cells from healthy controls with their autologous serum [371.9 (203.8–567.3) versus 239.7 (176.2–390.2); *p* = 0.0488] (Figure 7B).

The coculture of HMVEC-d with NK cells from SSc and control serum trended to result in more EMP release than NK cells from healthy controls with autologous serum [337.7 (249.7–518.6) versus 239.7 (176.2–390.2); *p* = 0.0742]. However, the addition of SSc serum to NK cells from healthy controls did not modify the HMVEC-d EMPs release. These findings suggested that the observed enhancement of EMPs release might be mediated by NK cells from SSc patients (Figure 7B). In this line, we observed that IL-6 levels in the supernatants of coculture NK cells from SSc patients and healthy controls were positively correlated with EMPs release (*r_S_* = 0.56; *p* = 0.0218).

In addition, we observed a trend to a negative correlation between the percentage of expression of CX3CR1 on NK cells and the enhancement of EMPs releasing triggered by NK cells from SSc patients and their autologous serum (*r_S_* = −0.6; *p* = 0.0968).

## Discussion

Our study evidenced quantitative and phenotypic features that characterize cytotoxic immune cells in SSc with a special focus on NK cells. Circulating CD8 T cells were decreased while circulating γδ T cells and NK cells numbers were preserved in SSc patients. Circulating NK cells from SSc patients exhibited decreased expression of CX3CR1 (fractalkine receptor), and CXCR4 (SDF-1 receptor) chemokine receptors, as well as of the NKG2D activating receptor and CD69 activation marker. DNAM-1 expression was similar in SSc patients and controls but inversely correlated with lung involvement assessed by DLCO in patients. CD16 expression was maintained in SSc patients analyzed in reference with controls. NK cells from SSc patients displayed preserved antibody-dependent degranulation ability compared with healthy controls and a trend to a decreased potential to exert natural cytotoxicity in the presence of their autologous serum. Interestingly, NK cells purified from SSc patients induced higher endothelial activation through microvascular microparticles release than healthy controls.

Our observation of decreased circulating CD8 T cells in SSc patients was consistent with previous reports (42–44). Other studies have also shown an increase of CD8 T cells in the bronchoalveolar fluid of SSc patients with interstitial lung disease (ILD) (45) which may suggest their recruitment to the lung where they would exert their cytotoxicity.

In the present study, SSc patients exhibited preserved numbers and percentages of NK cells and γδ T cells in their peripheral blood. Few studies have assessed NK cells and γδ T cells circulating compartments in SSc patients and reported discrepant results. Some studies found normal (43, 46), or decreased NK cells (47) and normal (48, 49) or decreased γδ T cells counts (50–52). These apparently conflicting results might partly result from differences in the gating strategy to define cell populations and/or characteristics of both patients and controls.

As we and others have previously identified soluble fractalkine to belong to the endothelial inflammation signature of SSc (17, 21), we investigated whether the expression of the CX3CR1 fractalkine receptor in cytotoxic immune cells could be affected in the context of enhanced release of this chemokine. Our analysis did not conclude to significant alterations in CX3CR1 expression in CD8 T cells and γδ T cells. Hasegawa et al. have previously described an increased expression of CX3CR1 in circulating CD8 T cells of SSc patients with diffuse cutaneous form compared with controls, whereas no difference was found between SSc patients with limited and diffuse cutaneous involvement (17). However, the low number of patients with the diffuse cutaneous form in our study (*n* = 4) may explain the lack of difference evidenced.

Strikingly, we observed a marked decrease in the percentages and expression level of CX3CR1 in NK cells of SSc patients. Hasegawa et al. have shown a conserved expression of CX3CR1 in CD16^+^ cells, defined as NK cells, but did not assess the other markers defining the NK cells subset, such as the lack of CD3 expression and the expression of CD56 (17). This difference of cytometry gating strategy of NK cells could explain the discrepancy between our data.

We observed a decreased expression of CX3CR1 in NK cells that might suggest its engagement with membrane-bound endothelial fractalkine and the recruitment of NK cells, chemoattracted by soluble fractalkine toward organ, through inflamed endothelium. Consistent with this hypothesis, Hasegawa et al. found increased CX3CR1 expression in inflammatory cells, colocalizing with CX3CL1 fractalkine-expressing endothelium in target organs such as lung and skin in SSc patients (17). Hence, we cannot rule out that soluble fractalkine levels are linked to CX3CR1 expression within SSc targeted tissues.

As demonstrated for NKG2D activating receptor, the decreased CX3CR1 expression on NK cells could rely on TGFβ, given that these two receptors have been demonstrated to be downregulated following TGFβ exposure (53, 54). Such event could be expected, as increased TGFβ signaling has been involved in the pathogenesis of SSc (55).

We noticed a rather high heterogeneity of CX3CR1 expression on NK cells in SSc patients. We thus further analyzed the variations in CX3CR1 expression levels within NK cells according to the clinical characteristics of patients and found a positive association with the pulmonary fibrosis and anti-topoisomerase 1 antibodies. Due to the low number of subjects in this study, no definitive conclusion can be drawn. Nevertheless, such observations suggest that the specific activation of the fractalkine/CX3CR1 pathway may favor NK cells-based mechanisms associated with the development of organ-specific involvement. Further investigations specifically addressing this point remain required in a larger cohort of patients.

We found no correlation between fractalkine levels and the level of expression of CX3CR1 in CD8 T cells, NK cells, and γδ T cells. Qualitative factors such as polymorphic variants of CX3CR1 could affect the binding affinity of CX3CR1 to fractalkine ligand. This may account for the lack of correlation between fractalkine and CX3CR1 observed here (18). However, the percentage of Vδ1 CX3CR1^+^ cells was inversely correlated with the level of circulating fractalkine. This may also suggest the recruitment of this tissular subset of γδ T cells toward target organs in SSc, such as evidenced in skin biopsies (48) and in the bronchoalveolar fluid of SSc patients (56).

The finding of decreased CX3CR1 expression NK cells in SSc patients led us to further characterize the expression of chemokine receptors and activation markers/receptors in this specific subset. We found decreased expression of CXCR4 but conserved expression of CXCR3, in comparison with circulating NK cells from healthy controls. CXCR4 expression and percentages of CXCR4 expressing cells were recently shown to be decreased in circulating monocytes, CD8 T cells, and B cells in SSc (57). In SSc, increased tissular expression of CXCR4 and its SDF-1 ligand have been identified in dermal ECs of SSc patients (58). Hence, this decreased CXCR4 expression might reflect chronic stimulation of this receptor by SDF-1 or SDF-1 driven migration of CXCR4^+^ NK cells toward skin tissue.

As chemokine receptors CX3CR1 and CXCR4 were decreased in SSc patients, we wondered whether the activating receptors CD16, DNAM-1, and NKG2D that participate in NK cells cytotoxicity were also modified. We observed that CD16 and DNAM-1 expression were not affected in SSc patients. However, DNAM-1 expression level was inversely correlated with the TLCO, reflecting ILD and/or PAH. This was consistent with a previous study that evidenced decreased DNAM-1 expression in circulating NK cells of SSc patients with ILD and PAH (47). Interestingly, we also found that NKG2D expression and percentage of NKG2D-expressing cells were decreased in SSc patients. Chronic exposure to membrane-bound or soluble NKG2D ligands can induce NKG2D down-modulation in NK cells. However, to date in literature, no information on NKG2D ligands expression in SSc is available to our knowledge. Here, our preliminary data found an increased relative expression of the ULBP3 transcript, NKG2D ligand, following HMVEC-d exposure to SSc sera (*data not shown*). This might suggest an increased release of ULBP3 by ECs in SSc patients and a subsequent negative feedback that would lead to the NKG2D internalization.

As NK cells from SSc patients exhibited decreased expression of cytotoxic receptor NKG2D, we aimed to precise their activation state assessing the activation marker CD69 and showed a decrease of CD69. CD69 was identified as an early activation marker but was more recently associated with tissue residency of CD56^bright^ NK cells (10). Thus, the decrease of CD69 expression in NK cells might reflect circulating NK cells exhaustion but does not preclude NK cells intra-tissular activation in SSc target organs.

Altogether, lowered expressions of CX3CR1, CXCR4, NKG2D, and CD69 point out a phenotypic signature of NK cells associated with SSc. We assume that this phenotype might impact the trafficking of NK cells subset and recruitment into the targeted organs. The decrease of chemokine receptors and CD69 might reflect the loss of tissue retention signals and might be associated with the dysregulation of NK cells localization and activation.

We further investigated whether such NK cell phenotype would impact NK cell natural and antibody-dependent cell-mediated cytotoxic function in the context of this autoimmune disease. NK cells from SSc patients displayed preserved ADCC compared with donors consistent with a similar CD16 expression profile among NK cells. The evaluation of cytolytic CD107^+^ granules release by NK cells revealed a slight decreased ability to exert natural cytotoxicity through perforin/granzyme pathway. This was consistent with the observation of Horikawa et al. who evidenced decreased NK cells cytotoxicity toward the erythroleukemic cell line K-562 (46). Using a microvascular dermal EC line, Sgonc et al. demonstrated that NK cells from SSc patients displayed anti-EC antibody-dependent cytotoxicity mediated by Fas/Fas ligand interaction but not by the perforin–granzyme pathway (59). Conversely, in our study, the lack of CD16 engagement in NK cells of SSc patients in the presence of SSc sera and HMVEC-d cell line (*data not shown*) did not argue for a major contribution of such cytotoxic mechanism. Our results rather indicate a potential role of the NK cells-endothelium cross talk in the tissue recruitment of NK cells, endowed with the ability to release paracrine factors driving endothelial activation.

In this line, we show that circulating purified NK cells from SSc patients induced higher endothelial activation through microvascular EMPs release than healthy controls. This was observed both with SSc serum and healthy control serum rather suggesting an intrinsic capacity of SSc NK cells to promote this effect. Interestingly, we observed that percentages of CX3CR1 expressing NK cells tended to negatively correlate with the enhancement of EMPs release. This observation suggests a potential implication of CX3CR1 in NK cells-mediated endothelial activation that should be implemented in a larger number of patients.

This greater NK cells-induced EMPs release might also involve IL-6 secretion given that IL-6 levels in coculture supernatants were correlated with EMPs release. IL-6 released by NK cells has been shown to be an early event in SSc associated with SSc onset (60) and IL-6 secretion was demonstrated to be correlated with EMPs release and vascular inflammation associated with coronary disease (61).

The EMPs can promote or aggravate pre-existing vascular dysfunction in cardiovascular diseases (30) and drive endothelial senescence in response to environmental stress (62). Our results suggest that EMPs generated in response to endothelial interactions with NK cells may contribute to the amplification of endothelial dysfunction and of stress-induced senescence processes favoring the progression of vasculopathy during SSc.

In conclusion, this study identifies a peculiar profile of the chemokine and activating receptors of NK cells in SSc, which may reflect NK cells involvement in the SSc pathogenic process. It also highlights the role of NK cells as a potent immune inducer of endothelial activation through vesiculation, with a potential implication of CX3CR1 in this mechanism.

We acknowledge the limits of this study, partly inherent to the low number of patients. However, it is the first one to demonstrate that purified NK cells from SSc patients have a higher potential to activate EMPs release in the presence of autologous SSc serum, thus providing a mechanistic hypothesis linking NK cells to endothelial activation in this disease. Further studies are needed to define the actors and mechanisms involved in the cross talk between NK cells and ECs and the consequence of such EMPs release.

## Ethics Statement

This study was carried out in accordance with the recommendations of French directives regarding Biomedical Research and “Comité de Protection des Personnes Sud Méditerranée.” The protocol was approved by the “Comité de Protection des Personnes Sud Méditerranée.” All subjects gave written informed consent in accordance with the Declaration of Helsinki.

## Author Contributions

AB, JM, FS, FDG, BG, and PP designed the study and wrote the paper. AB, BG, GK, KM, PR, and MR-G included patients. SM included healthy controls. AB, LL, CD, and SR performed experiments. AB collected and analyzed data. All of the authors have reviewed the manuscript for critical content and approved the final version of the manuscript.

## Conflict of Interest Statement

The authors declare that the research was conducted in the absence of any commercial or financial relationships that could be construed as a potential conflict of interest.
